# Program development using intervention mapping in primary healthcare settings to address elder abuse: A randomized controlled pilot study

**DOI:** 10.1002/brb3.2153

**Published:** 2021-05-04

**Authors:** Sonia Oveisi, LAR Stein, Forozan Olfati, Shima Jahed

**Affiliations:** ^1^ Metabolic Diseases Research center Medical Faculty Qazvin University of Medical Science Qazvin Iran; ^2^ Department of Psychology University of Rhode Island Kingston RI USA; ^3^ Social & Behavioral Science & Center for Alcohol & Addiction Studies School of Public Health Brown University Providence RI USA; ^4^ Department of Behavioral Healthcare Developmental Disabilities & Hospitals Cranston RI USA; ^5^ Nurse and Midwifery Faculty Qazvin University of Medical Science Qazvin Iran

**Keywords:** culture, elder abuse, healthcare, implementation, intervention mapping

## Abstract

**Background:**

Abuse of elderly women is of great concern and yet relatively little is known about interventions.

**Objectives:**

The aim of this study was to develop and test a culturally informed treatment, based on Intervention Mapping (IM), for primary healthcare settings. The intervention targets family members of elderly women and seeks to reduce elder abuse.

**Methods:**

*N* = 80 family members of elderly women were randomized to intervention or control. Elderly women completed assessment prior to randomization. Elder abuse was measured by self‐reported frequency of neglect, physical, psychological, and financial abuse in the last 2 months across 16 items. Intervention included 4 sessions, each under 1 hr. At 2‐month follow‐up, elderly women completed an assessment. Linear mixed modeling was used for analyses.

**Results:**

Significant reduction in frequency of psychological abuse and neglect was found in comparison to control, with trend effects for financial abuse (*F* = 127.12, *p* < .005; *F* = 95.4; *p* < .005; and *F* = 16.53, *p* < .07, respectively). Physical abuse was infrequent.

**Conclusion:**

This culturally tailored intervention reduced elder abuse. Given its basis in IM, it is well‐positioned for roll‐out and testing in a larger randomized trial to study adoption, implementation, and sustainability in practice settings.

## Graphical abstract

A culturally informed treatment, based on Intervention Mapping (IM), for primary healthcare settings was developed. The intervention targets family members of elderly women and seeks to reduce elder abuse. The intervention reduced psychological abuse and neglect. Physical abuse occurred at very low rates. Implementation and further testing in healthcare facilities are recommended.

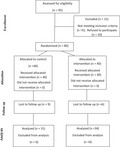

**Key Points**

- Intervention Mapping (IM) was used to develop a culturally informed treatment for primary care settings to address elder abuse.
- The intervention targets family members of elderly women.
- The intervention reduced psychological abuse and neglect. Physical abuse occurred at very low rates.

## 1 INTRODUCTION

Given the growth in the elderly population world‐wide (Strausbaugh, 2001; Yon et al., 2017), it is of utmost importance to maintain or improve quality of life (Ahrari et al., 2014; Feizabadi et al., 2016; Yaghoobzadeh et al., 2017). In some communities, high rates of abuse and neglect have been found among the elderly, with low social support frequently associated with increased risk of mistreatment (Acierno et al., 2010). Family members experiencing anxiety, depression, and stress (Anetzberger, 2000; Jane, 2003; Orfila et al., 2018; Wang et al., 2009) are at serious risk for abusing those ages 60 and older (Randel et al., 2017). In particular, family economic stress and poor elder health have been found to be associated with elder abuse (Mohseni et al., 2019). Lack of knowledge on what is considered abusive may also contribute to elder abuse and neglect (Khalili et al., 2016). The World Health Organization (Organization, 2002) defines elder abuse (EA) as “a single or repeated act, or lack of appropriate action, occurring within any relationship where there is an expectation of trust which causes harm or distress to an old person.” EA may include physical, psychological, sexual, and financial abuse, as well as neglect (John et al., 2012; Krug et al., 2002) resulting in shortened life expectancy in the elderly (Johnson et al., 2008). In most countries, prevalence of EA is estimated at 0.1%–10% (Acierno et al., 2010; Feltner et al., 2018; Laumann et al., 2008). The most common form of EA is neglect (Tolan et al., 2006).

Iranian elderly are not exempt from EA (Khanlary et al., 2016; Oveisi et al., 2014), and prevalence rates have been estimated at 48.3% (Abdi et al., 2019). This is noteworthy given that Islam, a dominant religion in Iran, indicates that elders should be respected (Hosseinkhani et al., 2017). Some research has found that a majority of victims are women (Yon et al., 2017). Elderly women often do not report EA due to fear of their families (Perel‐Levin & Organization, 2008). It may be that in countries with more traditional gender roles, including Iran (Darvishpour, 2002), elderly women are in greater need of programs to address EA as compared to men. Elders in Iran are routinely seen in health centers for medical care; however, EA interventions are not regularly provided for these patients and their families. Such a program should be provided, taking into consideration the family, community, and cultural contexts of Iranian elderly women (Abdi et al., 2019).

Behavioral theories, which commonly address coping skills, have been applied to understanding family conflict since the 1960s (Falloon, 2015) and have been used to design intervention programs beginning in the late 20th century (Baker, 1977). Social Learning Theory (SLT) (Bandura, 1977) has been used to understand complex human behavior (Grusec, 1994), including aggressive behavior (Anderson & Kras, 2005) and health behaviors (Chen et al., 2015). In brief, personal (e.g., self‐efficacy, motivations, expectations, values, and knowledge) factors, environmental factors (e.g., stress, supports, rewards, instruction, models, and encouragement), and behavior (e.g., abuse and caregiving) interact to influence each other. Primary, secondary, and tertiary social‐learning‐based intervention programs have been effective (Teresi et al., 2016) using multidisciplinary and collaborative approaches (Reis & Nahmiash, 1995) with a wide range of family problems and for culturally and economically diverse families (Biglan, 1995, 2016). Primary (universal) intervention programs do not require that an individual be at risk or show any signs of disorder. An advantage of universal programs is that no selection procedures are needed and thus stigmatization is unlikely to result (Dadds, 2002; Ganser et al., 2017; Hinsliff‐Smith et al., 2017; Holzer et al., 2006). Although universal interventions are strategies that target whole communities, it is important to tailor them to family needs and context.

In Iran, the group is considered before individual needs (Hofstede et al., 2005; Pourjalali & Meek, 1995). Family loyalty is important, and families may be more private than in many other cultures (CGC, 2020). Men are primary providers with few women working outside; and if elderly persons cannot support themselves, children do so, with kinship networks often serving as a primary support structure, especially in times of economic and social need (Madanipour, 2020). Developing an intervention for EA must account for willingness of families to reflect on behaviors, and the potential of kinship networks to assist some families in alleviating stress that can come with caregiving for an elderly person. The city in which the present study was conducted (Tehran) has a rising cost of living; noteworthy, unemployment and travel across the city are difficult (Madanipour, 2020). Therefore, an EA intervention must account for these practical considerations in terms of being relatively brief and not suggesting supports that will be costly. As compared to persons from other cultures, Iranians may be relatively less tolerant of ambiguity (Hofstede, 1980), and therefore a structured intervention (e.g., providing a preview and explanation for session activity) for EA may be better received as compared to an unstructured, un‐manualized approach. Several features of communication style in Iran also inform intervention design including importance of respect, avoiding embarrassment, and use of examples or stories to convey a point (Evason, 2016). Therefore, normalizing family struggle would be important and use of hypothetical examples to discuss difficulty may be in order as families consider hurtful behavior and alternatives (Oveisi et al., 2020). While it is important to design intervention with general contextual trends in mind, it is of utmost important to meet families where they are as individual units. Families have their own histories, knowledge, and habits, and indeed, many of the above considerations could apply across countries.

Intervention Mapping (IM) represents an ideal approach to develop an intervention for EA and has been used for decades in health settings [e.g., (Bartholomew et al., 1998; Cullen et al., 1998; Merlin et al., 2018)]. IM is well‐suited to the current task in that health interventions often require integration of multiple sources (e.g., literature, theory, data collection, expert consult) in planning. IM is a planning framework providing a systematic process and protocol for effective decision‐making for intervention development, implementation, and evaluation. An ecological approach is used to understand health problems and intervene on multiple levels (e.g., individual, family, setting). It emphasizes use of theory (such as SLT) and evidence (such as survey data); and attends to intervention deployment strategies (e.g., fit with setting needs), person–environment factors to effect change, and packaging of such factors into a coherent intervention. See (Fernandez et al., 2019).

The aim of this study was to investigate the impact of a family intervention package in primary healthcare settings to engage and intervene with family members of elderly women. Hypothesis: Elderly women suffering abuse will report a reduction in EA frequency. The study is important for several reasons: (a) A critical societal problem is addressed using a culturally tailored approach developed with Intervention Mapping (IM). IM is a strategy to develop theory‐ and evidence‐based interventions specific to population needs (Eledge et al., 2011) that encourages adoption, implementation, and sustainment of intervention (Fernandez et al., 2019) by organizations in practice settings. (b) Few interventions have addressed caregivers and yet such interventions may be effective (Pillemer et al., 2016), especially if coping skills are addressed (Baker et al., 2017). (c) Methodological weaknesses (e.g., small N) limit conclusions of prior EA research (Baker et al., 2017; Burnes et al., 2020); however, the current study improves on methodology (e.g., randomization, blinding, manualized intervention, and larger N).

## 2 METHODS

### 2.1 Design and procedures

This randomized controlled pilot study was conducted in primary healthcare centers (*N* = 2) located in Qazvin province from August to December 2017. Convenience sampling was performed based on the number of elderly persons receiving medical services. Sample size calculation (*N* = 80 elderly women) was based on a study conducted in 2014 (Alon & Berg‐Warman, 2014) in which α = 0.05, β = 0.2, p_1_ = 70% (incidence of psychological abuse before intervention) and p_2_ = 50% (incidence after intervention). Inclusion criteria: Being an elderly woman, 60 years or older; willing to participate in initial and follow‐up assessments; oriented to time and place; ability to respond to assessment procedure; and a family member willing to participate. Exclusion criteria: Stated unavailability during study follow‐up interview; and family member failure to participate in all intervention sessions. Family member inclusion criteria: Willing and able to participate in intervention; and oriented to time and place. Exclusion criteria: Failure to participate in all intervention sessions. If multiple family members met criteria, the member spending the most time with the elderly participant was preferred. Note that families were purposely not screened in based on EA, as the study is concerned with both primary and secondary intervention and prior work in these settings indicates high incidence of abuse (Abdi et al., 2019; Khanlary et al., 2016; Oveisi et al., 2014).

Advertisements were placed in healthcare centers, and *N* = 95 elderly women expressing interest were screened face‐to‐face. Fifteen did not meet inclusion/exclusion criteria leaving 80 women who were consented and whose family members (*N* = 80) were randomized to intervention or control groups using Balanced Block Randomization method (AABB, ABBA) with 20 blocks. Prior to randomization, baseline assessment was completed with elderly participants. Two months following final intervention session, follow‐up assessment was conducted with elderly participants. Research staff collecting data were blind to condition, participants were unaware of which condition was considered active versus control, and during analyses, treatment condition was masked (i.e., analyst was blind to group). Staff collecting data were trained and supervised by a doctoral‐level professional versed in data collection.

### 2.2 Participants

Eighty elderly women age 60 years or above receiving healthcare services from health centers in Tehran, Iran, participated, along with *N* = 80 family members. The study was approved by the ethics committee of Qazvin University of Medical Sciences, and written informed consent was utilized.

### 2.3 Outcomes

Main outcomes were the frequency of physical, psychological and financial abuse, and neglect as reported by elderly participants. See measures description below.

### 2.4 Materials

The design of the intervention package was based on Intervention Mapping (IM), a stepwise process for systematic development and evaluation of theory‐ and evidence‐based interventions targeted to population needs (Eledge et al., 2011). There are six steps in IM (Fernandez et al., 2019):

1. Needs assessment, specifying what needs to be changed and for whom. A survey provided to elderly patients (*N* = 600) of health centers in Tehran determined noteworthy incidence of psychological and financial abuse and neglect, some occurrence of physical abuse and very infrequent reports of sexual abuse. Family members of elderly participants in the current study completed a short questionnaire on key determinants of EA (e.g., stress) and the definition of EA. These surveys and the literature review above indicated EA should be targeted via intervention with caregivers.
2. Identification of behavioral targets for change. Targets included caregiver knowledge, supports, and coping skills, each which can influence EA. These were determined via the needs assessment in step 1.
3. Identify theory‐ and evidence‐based behavior change methods targeting outcomes, and translate these into practical applications for the intervention context. SLT is the basis of change methods used; and to enhance practicality, intervention design accounts for healthcare setting (e.g., efficiency of brief group sessions) and sample needs (e.g., structured sessions; see literature review above). Because targets as specified in Step 2 (above) involved family members, elderly persons were not included in group sessions. This made groups smaller, more manageable, and was meant to encourage attendee candor. Knowledge was addressed via pamphlets and educational didactics. Coping skills were addressed by improving self‐efficacy; however, this was first facilitated by enhancing interest in behavior change. Interest was fostered by examination of personal values regarding respect for the elderly and caring for them, in comparison to current personal behavior toward them; and by examining pros/cons of behavior (Miller & Rollnick, 2012). Respect was conveyed by collaborating with family members on what goals, if any, they may wish to set around their interactions with elderly persons. Efficacy was enhanced via group facilitator encouragement, modeling, and instruction; and via structured exercise where group members considered what might bolster confidence in behavior change. Hypothetical scenarios were provided to elicit group discussion around identification of functional and less functional behaviors in various situations including communication with an elderly relative, or methods of self‐care to reduce stress (e.g., perhaps seeking family or other supports as available). Group members were encouraged to provide support and suggestions to each other as appropriate. To solidify benefits of participation, previous topics were reviewed, role‐plays were used, and challenges were identified along with potential solutions. Members were asked to imagine the potential positive impact they may have on their families and communities going forward, and were thanked for participation.
4. Combine the intervention package into a coherent whole. Components of Step 3 above were organized into four sessions, each under 1 hr (Table 1). Sessions were didactic and interactive. A manual was developed with information based on literature review (e.g., as noted above, caregiver stress is a risk for EA) and exercises (e.g., exploring pros/cons of behavior change) based on the research team's prior work [e.g., (Oveisi et al., 2020)]. Sessions progressed from informational, to self‐examination, to enhancing coping, to solidifying change efforts. Feedback was obtained from a healthcare provider on materials and revisions made prior to this pilot study.
5. Develop or find implementation strategies to facilitate program adoption, implementation, and sustainability. An important strategy to encourage intervention dissemination is to involve professionals who will use the intervention in their intended settings (Bartholomew et al., 1998); this pilot indeed took this approach. To encourage adoption, implementation, and sustainment, efficient use of space is needed in busy healthcare settings. Therefore, sessions were on‐site, under 1 hr, were manualized and group‐based. Expectation of program success also facilitates adoption and sustainment, and data from the current pilot trial are intended to support such expectation for later dissemination. Additional implementation strategies (Waltz et al., 2015) incorporated into this pilot included use of academic partnership (i.e., Qazvin University), availability of a local champion (SO), use of clinical team meeting (to support interventionist), staging of scale up (i.e., pilot work), and tailoring (based on culture).
6. Plan a process and outcomes evaluation. The evaluation focused on EA outcomes as reported by elderly participants. Process evaluation (e.g. change in family member knowledge) was de‐emphasized as the goal was to demonstrate meaningful improvement on primary outcomes in this study, in order to garner evidence for an expanded implementation/dissemination study. Stakeholders are often more concerned with ultimate outcomes (i.e., EA) in any event. The measure used to evaluate EA is based on prior empirical work (see below). To rigorously evaluate the intervention, randomization was used with testing conducted at pre‐intervention and follow‐up at 2 months following last intervention session. This 2‐month period is adequate to demonstrate behavior change, although longer follow‐up may be used to demonstrate behavior maintenance (Velicer et al., 2000).

**TABLE 1:** The intervention sessions

Sessions	Content of sessions	Duration of session
First session	Introduction Program introduction (e.g., number and length of sessions, source of material, ground rules for group sessions) Defining EA concepts (e.g., types of EA, such as physical abuse), risk factors (e.g., familial stress), and consequences (e.g., personal, societal) Setting the time of the next session, eliciting commitment to attend coming session	45 min
Second session	Giving information on common processes associated aging (e.g., sight, mobility) Definition of different types of EA (e.g., financial abuse can involve controlling someone's money without permission) Encourage re‐evaluation of potentially abusive behavior (e.g., How would you know if your behavior were abusive or not?) and exploration of values (e.g., What are your values around taking care of elderly persons and why?). How does current behavior fit or not with values? Examining risks of the current behavior (e.g., on elderly, on modeling for children, potential legal problems, on community) What, if anything needs to change? Defining goals Setting the time of the next session, eliciting commitment to attend coming session	45 min
Third session	Personal/interpersonal skills (e.g., healthy communication with the elderly; self‐care) Evaluating advantages/disadvantages of changing behavior or not. Reminder of goals set in session 2 Enhancing confidence to change (e.g., On a scale from 0 to 10, where 0 = not confident to 10 = confident, how confident are you that you could change behavior if you wanted? Why are you an *X* instead of an *X*–1? What would it take to get you to be an *X* + 1?) Group members assist each other in problem‐solving barriers to change Setting time for next sessions, eliciting commitment to attend next session	45 min
Fourth session	Review of previous topics: Group members asked to recall session topics (e.g., EA definitions, risk factors, consequences, aging process, communication, values, goals, problem‐solving barriers to change), and given encouragement for trying Provide pamphlets for other family members with information and resources Role‐plays for new behaviors How to maintain the new behavior (reward self for meeting goal, seek social support) Thanking the families; honoring courage and engagement. This is an opportunity to help elderly, group members to help themselves and their communities	40 min

Abbreviations: EA, Elder abuse; Min, Minutes.

### 2.5 Measures

A self‐report questionnaire (Oveisi et al., 2014) was used to collect demographic information from elderly participants such as age, marital status, relation to primary caregiver, level of education, medical disease status (i.e., primary reason for receiving health services at clinic), income, home ownership, and EA. EA was assessed with 16 questions scored on a Likert scale: Never = 0, Once = 1, Twice = 2, Three times = 3, Four times = 4, Five times = 5, Six times = 6, Seven times or more = 7. Questions assessed neglect (sample item, “Have you been lonely?”) and physical (e.g., “Has anyone tried to hurt you or harm you?”), psychological (e.g., “Has someone screamed or yelled at you?”), and financial (e.g., “Have you been asked to sign papers you did not understand?”) EA. Cronbach's alpha is 0.77 in the current study based on standardized items (see Table 2).

**TABLE 2:** Elder abuse questions^a^ detecting four types of abuse. Within the past 2 months, how many times…

**Physical abuse**
1. Have you been hit, kicked, punched, or otherwise by someone?
2. …Has anyone close to you tried to hurt you or harm you?
**Neglect**
3. …Were you sad or lonely?
4. …Have you been hungry?
5. …Have you been in conditions in which you needed help and ask for help, but were ignored by your family members?
6. …Have you been in a situation where you were scared at home?
7. …Have you had thoughts of taking your life, even if you would not really do it?
**Financial abuse**
8. …Has anyone taken things that belong to you without your approval?
9. …Have you been forced to give cash to your family members?
10. …Have you been asked to sign papers you did not understand?
**Psychological abuse**
11. …Has anyone forced you to do things you didn't want to do?
12. …Have you experienced living in fear because somebody has threatened you?
13. …Has anyone close to you ever completely refused to talk to you or ignored you for days at a time, even when you wanted to talk to them?
14. …Have you been verbally threatened or insulted by others?
15. …Has someone screamed or yelled at you?
16. …Have you been afraid of your family members?

^a^ Response options from Never = 0 to Seven times or more = 7. Self‐report questionnaire provided to elderly women participants (see Measures).

### 2.6 Treatment and control groups

The interventionist was a woman experienced in nursing and midwifery. Training and supervision were provided by a PhD‐level professional also trained in midwifery and nursing. During an initial face‐to‐face contact with the interventionist, family members in the control group received general information on non‐communicable diseases (e.g., Diabetes, Blood Pressure), were advised to seek resources should they feel they may be needed, and were provided with a list of resources. As is usual care, all elderly women reporting abuse received brief education on elder abuse and information on local resources. Active intervention consisted of four consecutive sessions performed once per week in group format (see Table 1). Number of family members per group was *N* = 10, with four separate groups conducted. All participants, regardless of group, received education about elder abuse if detected.

### 2.7 Statistical analysis

Descriptive statistics were calculated and are presented for the sample in terms of Mean (*M*), Standard Deviation (*SD*), frequency (*n*), and percent (%). Data were examined to determine whether they conformed to distributional assumptions using Kolmogorov–Smirnov Test. Data not meeting distributional assumptions were analyzed using Linear Mixed Models, controlling for initial levels of outcome variables. For comparing categorical variables, *X* ^2^ was used. Level of significance was set at 0.05. Also, we calculated Cohen's *d* effect size (ES) based on the general guidelines of Cohen's *d* which are Small (0.2), medium (0.5), and large (0.8) for interpreting the effect of an intervention.

### 2.8 Ethics approval

Medical Research Ethics Committee of the Qazvin University of Medical Science approved this study on November 8, 2016 (IR.QUMS.REC.1395.184). Participants provided written informed consent for the study. This study was registered at the Iranian Registry of Clinical Trials (IRCT2017061234496N1).

## 3 RESULTS

**FIGURE 1:**
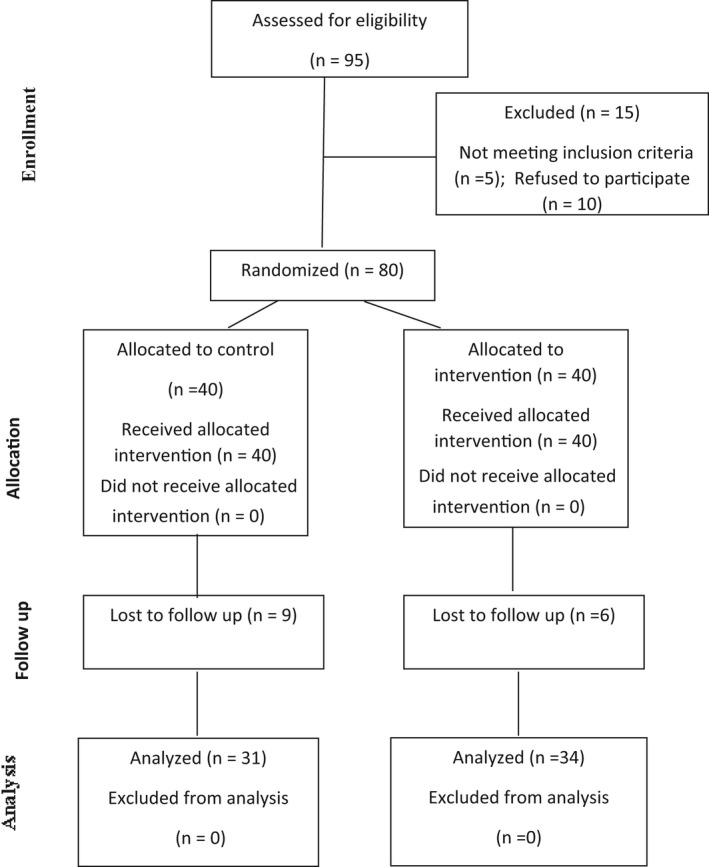
The CONSORT diagram showing the flow of elderly participants through each stage of a randomized trial

Loss to follow‐up was 6 from the intervention and 9 from the control conditions (see Figure 1). Age is described as follows: Control condition, 60–89 years, *M* = 69.16, *SD* = 7.43; intervention condition, 60–95 years, *M* = 70.55, *SD* = 8.56; with *t* test = 48.0, *p* =.69. Table 3 shows demographic information for elderly women (e.g., education level, marital status) with no differences between intervention groups. Elderly women lost to follow‐up did not differ statistically significantly from those retained at follow‐up.

**TABLE 3:** Comparing demographic characteristics of elders across control and intervention groups

Characteristic	Intervention group (*N* = 34, Total)		Control group (*N* = 31, Total)		Chi‐square	*p*‐Value
	*N*	%	*N*	%		
Elders’ education						
No years of education	28	82.4	24	77.4	3.61	.65
High school and less	5	14.7	6	16.1		
College and more	1	2.9	2	6.5		
Elders’ marital status						
Married	22	64.7	21	67.7	79	.06
Single	12	35.3	10	32.3		
Elders’ diseases						
Cardio‐pulmonary disease	6	17.7	5	16.1	1.93	.92
Diabetes	10	29.4	11	35.5		
High blood pressure	11	32.4	10	32.3		
Miscellaneous	7	2.5	5	16.1		
Primary caregiver						
Husband	19	55.9	21	67.7	1.37	.5
Children	7	2.6	6	19.4		
Nobody	8	23.5	4	12.9		
Status of home ownership						
Owned	34	100	30	96.8	1.11	.29
Rented	0	0	1	3.2		
Level of income						
Low^a^	13	38.2	12	38.7	9.8	.99
Moderate & high	21	61.8	19	61.3		

Note

Data collected via self‐report from elderly women participants (see Measures section).

Abbreviations: *N*, number; %, percent; *p*, significance level.

^a^ Below, at or marginally above poverty.

Table 4 shows *M*’s and *SD*’s on scores for neglect as well as financial, psychological, and physical abuse. In the intervention group, neglect and psychological abuse (*M* = 2.1 [*SD* = 2.29], *M* = 3.23 [*SD* = 3.34], respectively) were significantly reduced (*p* < .005, for both) at 2‐month follow‐up assessment compared to control group (*M* = 1.58 [*SD* = 2.14], *M* = 3.50 [*SD* = 3.40], respectively), with a non‐significant trend (*p* = .07) for less financial abuse in the intervention condition (*M* = 0.49 [*SD* = 1.15]) compared to control (*M* = 0.76 [*SD* = 0.23]). Physical abuse was reported at very low levels in both conditions (Range: *M* = 0 [*SD* = 0] to *M* = 0.03 [*SD* = 0.17]), and there were no significant differences at follow‐up (*p* = .13).

**TABLE 4:** Comparing the frequency^a^ of four types of elder abuse in control and intervention groups using linear mixed models

Elder abuse	Group	Time	Mean ± *SD*	Min	Max	*p*‐Value	*F*‐test
Neglect	Intervention	Before	2.77 ± 2.9	0	10	<.005	95.4
		After	2.1 ± 2.29	0	9		
	Control	Before	1.55 ± 2.16	0	7		
		After	1.58 ± 2.14	0	7		
Financial abuse	Intervention	Before	0.57 ± 1.42	0	7	.07	16.53
		After	0.49 ± 1.15	0	7		
	Control	Before	0.76 ± 0.23	0	5		
		After	0.76 ± 0.23	0	5		
Psychological abuse	Intervention	Before	4.4 ± 4.44	0	21	<.005	127.12
		After	3.23 ± 3.34	0	16		
	Control	Before	3.48 ± 3.6	0	14		
		After	3.5 ± 3.4	0	14		
Physical abuse	Intervention	Before	0 ± 0	0	0	.13	2.29
		After	0 ± 0	0	0		
	Control	Before	0.03 ± 0.17	0	1		
		After	0.03 ± 0.17	0	1		

Note

Data collected via self‐report from elderly women participants (see Measures); Table 2 also has items comprising each Elder abuse type.

Abbreviations: Max, Maximum; Min, Minimum; *p*, significance level; *SD*, Standard Deviation.

^a^ Frequency was coded as follows: Never = 0, Once = 1, Twice = 2, Three times = 3, Four times = 4, Five times = 5, Six times = 6, Seven times or more = 7.

Results showed that effect size for neglect, financial abuse, and psychological abuse was 0.23, 0.06, and 0.26 respectively. This means that the difference between financial abuses of two groups' means is <0.2, so, the difference is negligible, even if it is statistically significant. Also, it indicates the necessity of larger sample sizes.

## 4 DISCUSSION

The present study was conducted to examine the effects of an intervention for family members to prevent and reduce abuse of elderly women. Results show the intervention, based on Intervention Mapping (IM), can reduce elderly women's reports of neglect and psychological abuse. Several studies suggest abuse may be reduced by identifying family needs, decreasing family stress, and enhancing communication (Ganser et al., 2017; Hinsliff‐Smith et al., 2017; Newman, 2017). Innovative approaches are needed, such as development of caregiver‐based programs for elder abuse (EA), especially since caregivers are rarely a focus of such programs (Ploeg et al., 2009), and yet such approaches may be promising (Pillemer et al., 2016). In particular, a Cochrane review indicated that teaching coping skills to family caregivers of elderly persons with dementia may reduce risk of abuse (Baker et al., 2017). EA interventions should be tailored for cultural relevance (Dong et al., 2013), and few such programs have been developed and tested in Iran (Khanlary et al., 2016).

One study conducted in Iran provided families (*N* = 27) with five sessions of home‐based cognitive behavioral intervention delivered by a social worker and found less psychological and financial abuse, but no change in physical abuse, at follow‐up as compared to control (Khanlary et al., 2016). Similarly, six unstructured family counseling sessions (each 1.5–2 hr) were provided to Iranian elderly women (*N* = 30) and their families, with results indicating reduced psychological abuse at 2‐month follow‐up (Heravi Karimoi et al., 2005). Results of these studies are consistent with those presented here. However, the present study is briefer; had larger sample size; used rigorous methodology; and was designed to account for culture, and expressly to enhance implementation and dissemination.

Limitations include lack of process measures (e.g., family change in support, efficacy) to inform mechanisms of action. Future work should include family process measures and organizational measures including perceptions of intervention usefulness, penetration of the intervention, and costs to sustain the intervention. Because this was a pilot, sample size was not large enough to test nesting within setting or group and future studies may wish to examine this. Although formal fidelity tool was not used, regular supervision of manualized intervention reduced contamination and assisted in maintaining intervention integrity. Contact time between intervention groups was not controlled; however, it is useful to understand if an improvement can be made over standard care. Future work can control for contact time. Although sex abuse was not studied, it has been found to occur at very low rates (Oveisi et al., 2014); future studies can include this form of EA. Future work must include longer follow‐up which may enhance ability to examine intervention effects for prevention (especially for physical abuse). Similarly, larger sample size in future work may assist in reaching significance for some trending differences in the current study (i.e., financial abuse). Other details can be collected in future studies including perpetrator (family or other person), and impact of intervention by abuse type and family member type (son, daughter, spouse). This intervention targeted elderly women with an identified family member willing to participate, and as such may not be of assistance to elderly women who are more isolated. Women who are isolated and experiencing EA may benefit from other forms of intervention including those emphasizing community outreach.

## 5 CONCLUSIONS

Findings indicate a culturally informed approach, based on Intervention Mapping, for family members can reduce psychological abuse and neglect of elderly women. Results were promising for financial abuse, although not statistically significant. Physical abuse occurs at relatively low rates. The intervention package is designed to fit well into healthcare settings in Iran and to address families at primary and secondary intervention levels. Therefore, it may be useful to roll it out more completely and test effectiveness in a larger randomized trial in order to address this important public health concern.

## CONFLICT OF INTEREST

None declared.

## AUTHOR CONTRIBUTIONS

(a) SJ, FO, and SO involved in concept and design; (b) SJ involved in pre‐intervention data collection and intervention delivery; (c) SO involved in data analysis; (d) FO, SO, and LS involved in interpretation of data; (e) LS is expertise in implementation science, behavioral interventions and cultural adaptions; (f) FO, SO, and LS drafted the article; (g) SO and LS revised the article for important intellectual content; (h) SO finally approved the version to be published. All authors have read and approved the manuscript.

## CONSENT FOR PUBLICATION

Not applicable.

### PEER REVIEW

The peer review history for this article is available at https://publons.com/publon/10.1002/brb3.2153.

## Data Availability

The datasets used and/or analyzed during the current study are available from the corresponding author.
